# Calcium intake: good for the bones but bad for the heart? An analysis of clinical studies

**DOI:** 10.1590/2359-3997000000173

**Published:** 2016-02-11

**Authors:** Guilherme Alcantara Cunha Lima, Priscilla Damião Araújo Lima, Maria da Glória Costa Reis Monteiro de Barros, Lívia Paiva Vardiero, Elisa Fernandes de Melo, Francisco de Paula Paranhos, Miguel Madeira, Maria Lucia Fleiuss de Farias

**Affiliations:** 1 Universidade Federal do Rio de Janeiro Rio de Janeiro RJ Brasil Serviço de Endocrinologia da Universidade Federal do Rio de Janeiro (UFRJ), Rio de Janeiro, RJ, Brasil; 2 Faculdade de Medicina de Campos Campos dos Goytacazes RJ Brasil Serviço de Clínica Médica da Faculdade de Medicina de Campos (FMC), Campos dos Goytacazes, RJ, Brasil; 3 Universidade Federal do Rio de Janeiro Rio de Janeiro RJ Brasil Serviço de Reumatologia da Universidade Federal do Rio de Janeiro (UFRJ), Rio de Janeiro, RJ, Brasil; 4 Centro Universitário Serra dos Órgãos Teresópolis RJ Brasil Serviço de Clínica Médica do Centro Universitário Serra dos Órgãos (Unifeso), Teresópolis, RJ, Brasil; 5 Universidade do Grande Rio Rio de Janeiro RJ Brasil Serviço de Clínica Médica da Universidade do Grande Rio (Unigranrio), Rio de Janeiro, RJ, Brasil

**Keywords:** Calcium supplementation, dietary calcium, osteoporosis, cardiovascular safety, cardiovascular mortality

## Abstract

The proper dietary calcium intake and calcium supplementation, when indicated, are important factors in the acquisition of peak bone mass during youth and in the prevention of fractures in old age. In addition to its deposition in bone, calcium confers an increase in its resistance and exhibits important activities in different enzymatic pathways in the body (e.g., neural, hormonal, muscle-related and blood clotting pathways). Thus, calcium supplementation can directly or indirectly affect important functions in the body, such as the control of blood pressure, plasma glucose, body weight, lipid profile and endothelial function. Since one publication reported increased cardiovascular risk due to calcium supplementation, many researchers have studied whether this risk actually exists; the results are conflicting, and the involved mechanisms are uncertain. However, studies that have evaluated the influence of the consumption of foods rich in calcium have reported no increase in the cardiovascular risk, which suggests that nutritional intake should be prioritized as a method for supplementation and that the use of calcium supplements should be reserved for patients who truly need supplementation and are unable to achieve the recommended daily nutritional intake of calcium.

## INTRODUCTION

**Figure 1:**
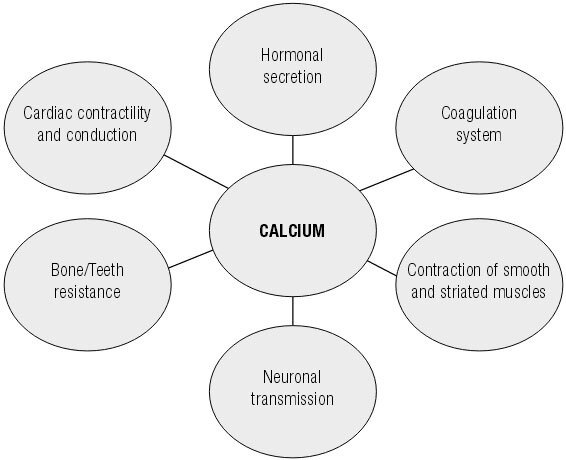
Calcium functions in the body.

Calcium plays an important role in the human body. Calcium mediates nervous excitability, muscle contractility, hormone secretion and blood clotting. Bones are the major calcium reservoir in the human body and holding 99% of the total bodily calcium. Calcium storage in the form of hydroxyapatite crystals provides rigidity to bones and minimizes the risk of fractures (1). Calcium is known to play important roles in the prevention and treatment of osteoporosis (2). However, the close relationships of calcium with myocardial contraction, nerve conduction, hormonal modulation and blood clotting may result in increased cardiovascular risk (3) (Figure 1).

In addition to confer resistance to bones and teeth, calcium plays an important role in several regulatory pathways in the body. It acts by modifying the activity of cellular receptors, in signal transduction and as a cofactor in various enzyme systems, thus modulating the secretion of hormones, such as insulin, mediating contractility of striated and smooth muscle, neuronal transmission and cardiac conduction besides being required in the intrinsic, extrinsic and common pathways of the coagulation system. The maintenance of serum calcium levels is required for the proper functioning of organism.

Indeed, patients with hypercalcemia exhibit increased mortality due to cardiac and vascular complications (4-6). Recent studies have evaluated the effects of medicinal calcium supplementation on cardiovascular risk and produced conflicting results (6-24) that have led to concerns about the safety of calcium supplementation and its role in the treatment of osteoporosis (25).

Studies that have evaluated the intake of calcium food sources have reported neutral or protective effects on major cardiovascular outcomes, such as atherosclerosis (26-28), blood pressure (29), diabetes (30,31) and body weight (32) and the risks of infarction (15,24), stroke (21,23,24) and cardiovascular mortality (6,7,24). Moreover, such food sources result in the minimization of the risk of nephrolithiasis (33), which is known to be increased by medicinal calcium supplementation (34), and these food sources can provide other nutrients, such as proteins, which are useful in maintaining bone mass in adults (35,36) and can minimize the risks of malnutrition and sarcopenia, which are indirectly related to bone quality and the risk of fractures (37). Thus, calcium intake from dietary sources should be a priority, and supplements should be reserved for patients who are at a real risk of osteoporotic fractures and who cannot achieve their daily nutritional needs. Drug overdoses must be avoided because transient hypercalcemia might be associated with increased cardiovascular risk (25,35,38).

The current review aims to analyze the importance of calcium in bone integrity and to discuss the main studies that have evaluated the cardiovascular safety of calcium supplementation.

## MATERIAL AND METHODS

During the periodo between 07/15/2015 and 09/18/2015, we reviewed all studies published from 1990 and 2015 on this subject in the PubMed and Capes portal databases. The following terms were searched: calcium supplementation and bone health, calcium supplementation and fractures, calcium supplementation and mortality, calcium supplementation and cardiovascular risk, calcium supplementation and hypertension, calcium supplementation and diabetes, calcium supplementation and obesity, and calcium supplementation and nephrolithiasis. Only studies published in English were considered. Among all of the pre-selected publications, we prioritized systematic reviews, meta-analyses, randomized controlled trials (RCT), longitudinal studies and recent publications.

### Calcium and bone health

Osteoporosis is characterized by decreased bone mass and microarchitectural deterioration that results in an increased risk of fracture (39). Although this disease is primarily diagnosed in the elderly, osteoporosis prevention should begin in childhood and adolescence via the adoption of behaviors that are aimed at the proper acquisition of peak bone mass and the prevention of bone mass loss, such as engaging in physical activity, refraining from smoking and consuming alcohol, increasing sun exposure, and the consumption of calcium-rich foods (40).

Studies that have evaluated the influences of eating foods rich in calcium during childhood and adolescence on bone health have been conducted. A recent double-blind RCT evaluated 220 teens after two years of low, medium or high calcium intake levels (through fortified milk) and found an increase in the bone mass of the female adolescents who ingested more calcium (41). Women who drink less milk during childhood and adolescence exhibit lower bone mass and an increased risk of fracture as adults (42).

The intake of calcium and its correlations with bone mineral density (BMD) and fractures in adults have also been assessed. Recently, Kim and cols. (43) evaluated 7,260 men and women aged ≥ 50 years and found that calcium intakes < 400 mg/day were correlated with lower BMD in the lumbar spine, lower femoral cortical thicknesses (in both genders) and lower BMD in the femoral neck (only in women). In contrast, intakes greater than 1,200 mg/day were positively correlated with BMD in the lumbar spine and femoral neck (only in men). Elderly people with higher milk or milk and yogurt intakes at baseline have been found to be a lower risk of hip fractures when assessed 10 years later (44). A reduced risk of fractures (30%) in subjects with higher nutritional calcium intakes was also recently described by Khan and cols. (24). Moreover, Key and cols. (45) reported an increased fracture risk in women with daily calcium intakes below 525 mg/day.

The effectiveness of isolated calcium supplementation in the prevention of osteoporotic fractures remains controversial. A meta-analysis involving 170,991 women and 68,606 men found that isolated calcium intake was not associated with a reduced fracture risk (46). Shea and cols. (47) evaluated the results from 1,806 participants and found that despite a BMD increase, isolated calcium supplementation did not reduce the risk of non-vertebral fractures. Similarly, the RECORD study evaluated the efficacies of supplementations with calcium, vitamin D or both in the elderly in the secondary prevention of low-impact fractures and concluded that none of these methods was effective in reducing the risk (48). However, Tang and cols. (49) published a meta-analysis that included 29 RCT and concluded that combined supplementation resulted in a reduction in the risk of fractures in patients aged 50 years or more. Another study reported a reduced risk of osteoporotic fractures and improvements in parameters related to bone density in women who consumed more than 80% of their calcium supplements per year over five years, which suggests that adherence to treatment is critical for the effectiveness of therapy (50). Low patient compliance with supplementation regimens due either to poor treatment adherence or gastrointestinal symptoms associated with calcium supplements may be one of the important determinants of these conflicting results (18,47-49).

Another factor that should be considered is that the intake of foods rich in calcium is not sufficient to meet the recommended dietary allowance (RDA) for most of the population. A chronic negative calcium balance may result in the stimulation of bone demineralization and negatively influence treatment efficacy. Heaney and cols. (51) reanalyzed six large studies and found that the calcium intakes of 85% of the participants were below the RDA. In our country, Pinheiro and cols. (52) reported the same problem and emphasized that the participants consumed only one-third of the daily calcium recommendation on average.

Combined calcium and vitamin D supplementation is classically recommended for the treatment of osteoporosis. Vitamin D deficiency may adversely affect bone mass by promoting reduced gastrointestinal calcium absorption and potentially promoting the hypersecretion of PTH, which stimulates bone resorption to restore the ionized calcium levels in the extracellular fluid (53). The ability to increase intestinal calcium absorption protects both youth and adults from bone demineralization. However, this ability becomes restricted in elderly people who need to use their body calcium to maintain homeostasis, which increases their risk of osteoporosis (40,54). Additionally, elderly individuals have lower levels of 7-dehydrocholesterol in their skin and are therefore less able to synthesize vitamin D from sun exposure, which may aggravate calcium imbalances and result in secondary hyperparathyroidism (55). Thus, inadequate vitamin D status can exacerbate the detrimental effects of low calcium intake, and vitamin D is therefore essential for the intestinal absorption of this ion at a level that is adequate to enable bone remodeling (35).

Kim and cols. (43) only observed a correlation between low calcium intake and reduced BMD in a subgroup with concomitant vitamin D deficiency, which reinforces the importance of vitamin D in conferring the benefits of calcium to the bones. Additionally, vitamin D supplementation leads to improved muscle strength, especially in the elderly (56), and reduces the risk of falls, which constitutes an additional factor in the prevention of osteoporotic fractures (57). Indeed, studies that have analyzed combined supplementation have observed the preservation of BMD, reductions of serum PTH and bone resorption markers (58), and reductions in the risk of fractures (2,49,59).

Calcium and vitamin D supplementation should not be generalized applied because individuals with low fracture risks need to be considered. Studies that have evaluated the effectiveness of indiscriminate treatment with calcium and vitamin D have failed to observe benefits regarding the risk of fractures because the individuals included in these studies exhibited very low risks of fractures and thus did not benefit from supplementation (34). Accordingly, the authors of a Cochrane review found that despite small increases in the BMD of healthy children, calcium supplementation was not able to reduce the risk of fractures, and these authors concluded that routine supplementation should not be recommended (60). Verbrugge and cols. (25) advocated calcium and vitamin D supplementation only for patients with documented risks of fractures, such as elderly patients, institutionalized patients, patients with diagnoses of osteoporosis and chronic glucocorticoid users. Furthermore, excessive supplementation above the RDA (Table 1) (61) should be avoided because it increases the risk of adverse effects and confers no additional benefit (25,35).

**Table 1:** Daily recommended calcium intake (Source: International Osteoporosis Foundation 2014 – Ref. 61)

Age/Gender	Calcium (mg/day)
Children/Adolescents	
0-6 months old	200
6-12 months old	260
1-3 years old	700
4-8 years old	1,000
9-18 years old	1,300
Female	
Pregnancy/lactation (14-18 years old)	1,300
19-50 years old	1,000
Pregnancy/lactation (19-50 years old)	1,000
Postmenopausal or ≥ 51 years old	1,200
Male	
19-70 years old	1,000
≥ 71 years old	1,200

### Calcium and cardiovascular risk

Given the frequent use of calcium supplementation for the prevention and treatment of osteoporosis in many patients, the safety profile of calcium supplementation and its potential interactions with other metabolic pathways that are related to the cardiovascular system have been tested in several studies (6-24) (Tables 2 and 3). Major concerns have been expressed following reports in some studies of increased cardiovascular risks associated with calcium supplementation (10-12,15,19).

Bolland and cols. (9) evaluated 1,471 postmenopausal women over 5 years and observed trends toward increased risks of myocardial infarction (MI) and stroke in the group that received calcium citrate. It is worth emphasizing that calcium supplementation was not administered with vitamin D in this study. The studied groups received high calcium doses (1,000 mg/day) that exceeded the RDA when the subjects’ average dietary calcium intakes were included (860 mg/day; Table 1). The same group of researchers then performed a meta-analysis of 16 studies that evaluated the cardiovascular outcomes of patients who received ≥ 500 mg/day of calcium and observed an increased risk of MI (31%); however, the risks of although stroke, sudden death, and MI + stroke + sudden death did not differ between the groups (11). This meta-analysis only evaluated studies with isolated calcium supplementation, which has resulted in criticisms of the design of this study. Bolland and cols. (12) subsequently published a new meta-analysis of 12 RCT (29,277 participants) that included the randomly selected subgroup of the WHI study that did not take calcium supplements and concluded that combined calcium and vitamin D supplementation increased the risks of MI and MI + stroke.

Other important studies have also reported increased MI risks in adult men and women (15) and an increased risk of death due to cardiovascular diseases in men (10,19) (Table 2). It is important to highlight that most of these studies were not designed to evaluate cardiovascular risk as a primary endpoint. The primary outcomes of these studies were the effects of calcium and/or vitamin D supplementation on fracture risk and not cardiovascular risk. Furthermore, the lack of standard criteria for the diagnosis of cardiovascular complications may have resulted in the under- or overestimation of the actual prevalences of these complications and thus resulted in possible bias in the results (25).

**Table 2:** Relationships between calcium supplementation and cardiovascular outcomes

Authors	Year	Number of participants	Characteristics of participants	Study design	Results
Bostick and cols. (7)	1999	34,486	55-69 years Post-menopausal women without previous ischemic heart disease (IHD)	Prospective cohort study Follow-up 8 years	No correlation between calcium supplementation and IHD death
Hsia and cols. (8)	2007	36,282	50-79 years Post-menopausal women WHI participants	Prospective RCT study Follow-up 7 years	No correlation between calcium/vitamin D supplementation and MI/stroke
Bolland and cols. (9)	2008	1,471	Post-menopausal women	Prospective RCT study Follow-up 5 years	Positive trends between calcium supplementation and MI/stroke
Pentti and cols. (10)	2009	10,555	52-62 years Women without CHD at baseline	Prospective cohort study Follow-up 7 years	Positive correlation between calcium/vitamin D supplementation and CHD (24%)
Bolland and cols. (11)	2010	20,072	Double-bind RCT studies > 40 years Calcium supplements	Meta-analysis (15 RCT studies)	Positive correlation between calcium supplementation and MI (27%) No correlation between calcium supplementation and stroke/sudden death/ MI + stroke + sudden death
Bolland and cols. (12)	2011	28,072	RCT studies Calcium or calcium/vitamin D supplements	Meta-analysis (9 RCT studies)	Positive correlation between calcium/vitamin D supplementation and MI (24%)/MI + stroke (15%)
Lewis and cols. (13)	2011	1,460	75 ± 2.7 years Women with preexisting atherosclerotic vascular disease	Prospective double-bind RCT study Follow-up 4,5 years	No correlation between calcium supplementation and atherosclerosis/general mortality
Avenell and cols. (14)	2012	5,292	≥ 70 years (women: 85%) Previous osteoporotic fracture	Prospective RCT study Follow-up 24-68 months	No correlation between calcium calcium/vitamin D supplementation and general mortality No correlation between calcium or calcium/vitamin D supplementation and vascular disease mortality
Li and cols. (15)	2012	23,980	35-64 years (women: 54%) Without major CVD events	Prospective cohort study Follow-up 11 years	Positive correlation between calcium supplementation and MI (86%) No correlation between calcium supplementation and stroke/general mortality
Rejnmark and cols. (16)	2012	70,528	Vitamin D or calcium/vitamin D supplements Women (86,8%) Median age: 70 years	Meta-analysis (8 RCT studies)	Negative correlation between calcium supplementation and general mortality (9%)
Langsetmo and cols. (17)	2013	9,033	≥ 25 years (women: 69.6%) Non-missing Calcium/Vitamin D intakes	Prospective cohort study Follow-up 10 years	Negative correlation between calcium supplementation + dietary calcium and general mortality (22%), independently of vitamin D intake
Prentice and cols. (18)	2013	46,892	50-79 years Post-menopausal women WHI participants	Prospective RCT study Follow-up 7 years	No correlation between calcium/vitamin D supplementation and MI/stroke/CVD death/general mortality
Van Hemelrijck and cols. (6)	2013	20,024	≥ 17 years (women: 52,5%) without history of heart disease NHANES III participants	Prospective cohort study	No correlation between calcium supplementation and CVD death
Xiao and cols. (19)	2013	388,229	50-71 years (Women: 43,5%) NIH participants	Prospective cohort study Follow-up 12 years	Positive correlation between calcium supplementation and CVD death in men (20%) No correlation between calcium supplementation and stroke in women No correlation between calcium supplementation and CVD death/stroke in women
Paik and cols. (20)	2014	74,285	30-55 years Women without history of CVD or cancer	Prospective cohort study Follow-up 24 years	Negative correlation between calcium supplementation + CVD (18%)/MI (29%) in women No correlation between calcium supplementation and stroke

RCT: randomized placebo-controlled trial.

In contrast, other studies have failed to find an association between calcium supplementation and the risk of cardiovascular complications (6-8,13,14,16-18,20) (Table 2). The initial assessments of the WHI participants revealed that medicinal calcium supplementation did not increase the risks of MI or stroke even in the subgroup with higher dietary calcium intake (8). However, separate subgroup analyses were not performed in this study; thus, individuals who did and did not use calcium supplements at the time of randomization were analyzed together, contrary to Bolland and cols. analysis (12), that includes only WHI patients who had no previous use of calcium supplements.

The WHI study participant data were re-evaluated after the publication of the study by Bolland and cols. (12) with a focus on whether the women participants were or were not using calcium supplements at the time of WHI enrollment, and no associations of calcium supplementation with increased risks of MI, stroke, coronary heart disease or death were observed in any of the subgroups (18). In an analysis of 20,024 National Health and Nutrition Examination Survey III (NHANES III) participants, no increase in cardiovascular diseases (CVD) mortality secondary to dietary or medicinal calcium intake was observed, although the risk of death from CVD was higher among patients with underlying hypercalcemia (6). A meta-analysis involving 8 RCT encompassing 70,528 participants with a mean age of 70 years concluded that calcium/vitamin D supplementation was associated with a 9% reduction in the risk of death from all causes (16). A recently published study analyzed 74,245 women over 24 years and found that calcium supplementation exhibited protective effects against cardiovascular and coronary heart diseases (CHD) and a neutral effect on stroke (20).

The influence of dietary calcium intake on cardiovascular outcomes has also been evaluated (6,7,15,17,19,21-24) (Table 3). A subgroup that ingested > 1,425 mg/day of calcium on average exhibited a reduction in the risk of CVD mortality of 33% compared with a subgroup with a calcium intake < 696 mg/day (7). Negative correlations of dietary calcium intake with MI (15,24) and stroke (21,23,24) risks have also described. Levitan and cols. (23) assessed the subgroup of WHI participants who were hospitalized due to heart failure over a period of 4.6 years (3,340 patients) and concluded that dietary calcium intake was not correlated with the risk of death. In a recent publication, Khan and cols. (24) demonstrated that the group with the highest dietary calcium intake (median of 1,348 mg/day) presented good cardiovascular safety profiles (a 14% reduction in mortality from all causes, a 16% reduction in non-fatal CVD and a 31% reduction in stroke) compared with the group with the lowest dietary calcium intake (median of 473 mg/day).

**Table 3:** Relationships between dietary calcium and cardiovascular outcomes

Authors	Year	Number of participants	Characteristics of participants	Study design	Results
Bostick and cols. (7)	1999	34,486	55-69 years Women post-menopausal without previous IHD	Prospective cohort study Follow-up 8 years	Negative correlation between dietary calcium and CVD death (37%)
Iso and cols. (21)	1999	85,764	34-59 years Women NHS participants	Prospective cohort study Follow-up 14 years	Negative correlation between dietary calcium and stroke (31%) in women
Li and cols. (15)	2012	23,980	35-64 years (women: 54%) without major CVD events	Prospective cohort study Follow-up 11 years	Negative correlation between dietary calcium and MI (31%) No correlation between dietary calcium and stroke/CVD death
Langsetmo and cols. (17)	2013	9,033	≥ 25 years (Women: 69,6%) Non-missing CaVit D intakes	Prospective cohort study Follow-up 10 years	Trends to negative correlation between dietary calcium and general mortality
Larsson and cols. (22)	2013	9,095	History of stroke	Meta-analysis (11 RCT studies)	Negative correlation between dietary calcium and stroke (22%)
Levitan and cols. (23)	2013	3,340	50-79 years Post-menopausal women WHI participants Heart failure hospitalization	Prospective cohort study Follow-up 4-6 years	No correlation between dietary calcium and CVD death
Van Hemelrijck and cols. (6)	2013	20,024	≥ 17 years (women: 52,5%) without history of heart disease NHANES III participants	Prospective cohort study	No correlation between dietary calcium and CVD death
Xiao and cols. (19)	2013	388,229	50-71 years (Women: 43,5%) NIH participants	Prospective cohort study Follow-up 12 years	No correlation between dietary calcium and CVD death/stroke
Khan and cols. (24)	2015	34,468	40-69 years (women: 60,3%) Without CVD, cancer and diabetes previous	Prospective cohort study Follow-up 12 ± 1,5 years	Negative correlation between dietary calcium and all-cause mortality (14%)/non-fatal CVD (16%)/stroke (31%)

### Calcium and cardiovascular risk: assumptions

The relationship between calcium supplementation and CVD risk and the manners in which calcium might protect or damage the cardiovascular system (62) remain inconclusive. The main hypothesis is that the sudden elevation of serum calcium levels after supplementation may result in increased vascular resistance and calcification as well as cardiac arrhythmias (3,9). Increased serum calcium levels have been observed 60-90 minutes after medicinal supplementation (63). However, Burt and cols. (64) evaluated endothelial function and myocardial perfusion parameters before and 3 hours after supplementation with 1,000 mg of calcium citrate and concluded that despite the elevated calcium levels, arterial constriction decreased, and myocardial perfusion increased; these results are suggestive of the cardioprotective effects of calcium. Slinin and cols. (4) reported a higher incidence of cardiovascular events among patients with hypercalcemia but failed to demonstrate that calcium intake was associated with increased baseline calcium levels in these patients.

Vitamin D insufficiency/deficiency is another possibility because it is highly prevalent among the elderly (i.e., the group with the greatest need for calcium supplementation) and has been consistently related to increased cardiovascular risk (65). Most studies have not assessed the levels of 25-OH vitamin D at baseline and after supplementation, which hinders their interpretations. However, because vitamin D insufficiency/deficiency is a highly prevalent disorder in several regions of the world across different age groups and socioeconomic classes (66), it is unlikely that a higher incidence of vitamin D deficiency in the intervention group compared with the placebo could account for this difference in cardiovascular risk (16).

Calcium supplementation does not seem to influence the emergence or worsening of hyperparathyroidism, which is a condition that is also associated with cardiovascular risk. A recent study found that calcium supplementation reduced the number of new cases of primary hyperparathyroidism among women (67). Another study found that calcium and vitamin D supplementation for 8 weeks reduced PTH serum levels by 17% (68). Calcium supplementation in patients with primary HPT resulted in reduced serum PTH levels in 17 of the 24 studied cases (69).

#### Calcium and endothelial function

Despite the higher prevalence of atherosclerotic plaques in the carotid arteries of patients with higher serum calcium levels (70), Lewis and cols. (71) found no increases in the risk of atherosclerotic plaques or carotid intima-media thicknesses in elderly women who received calcium supplementation for 3 years. The same group of researchers conducted a double-blind five-year RCT and found that calcium supplementation at 1,200 mg/day did not increase the mortality risk or the rate of hospital admissions due to atherosclerotic vascular disease (13). Another study found that the calcium coronary scores did not differ between women who underwent calcium and vitamin D supplementation for 7 years and a control group (72). Kim and cols. (73) also failed to observe an increased risk of calcification of the coronary arteries in patients with higher nutritional calcium intakes. Neither medicinal supplementation nor dietary calcium intake increased the risk of calcification in the coronary and carotid arteries or abdominal aorta of diabetic patients (26). Ivey and cols. (27) found that the intake of dairy products did not increase participants’ intima-media thickness. Additionally, yogurt intake was associated with a reduction in the thicknesses of these layers. Hyperhomocysteinemia (a marker of cardiovascular events in patients with atherosclerosis) is correlated with lower daily calcium intake (28), and not the reverse.

#### Calcium and blood pressure

The effect of calcium supplementation on blood pressure has also been evaluated (29,68,74-76). Wang and cols. (29) found a negative correlation between blood pressure and dietary calcium intake but found no correlation in a group that received supplementation. Pfeifer and cols. (68) found reductions in both systolic and diastolic blood pressures in patients receiving calcium (1,200 mg/day) and vitamin D (800 IU/day) supplementation. Another study examined 1,471 postmenopausal women and found a small decrease in diastolic blood pressure after 6 months of calcium supplementation that was not sustained after 30 months (74). The same author found no statistically significant differences between the blood pressures men who received calcium supplementation and men who received placebo treatment for 2 years (75). Another study also reported elevated systolic blood pressure related to calcium supplementation, although this finding was not clinically significant (76).

#### Calcium and diabetes mellitus

Studies have demonstrated beneficial or neutral effects of calcium dietary intake and medicinal supplementation on the development of diabetes (30,31,77,78). A meta-analysis involving 264,268 participants and 11,225 reported cases did not find a correlation between dietary calcium intake and diabetes (30). Tong and cols. (31) performed another meta-analysis and found an inverse correlation between the intake of dairy products and the development of diabetes. An analysis that assessed the role of medicines containing calcium in the risk of diabetes that was performed on the WHI study participants who received calcium and vitamin D supplementation for 7 years revealed no significant difference in newly diagnosed cases of diabetes compared with the control group (77). Calcium citrate and vitamin D supplementation for 3 years in patients with impaired fasting glucose has been found to result in attenuations of hyperglycemia and insulin resistance compared with placebo (78). Improvements in insulin sensitivity have also been reported in diabetic and hypertensive patients after 8 weeks of medicinal calcium supplementation compared with placebo (79).

#### Calcium and body weight

The effect of calcium supplementation on body weight seems uncertain. One study found neutral effects of calcium supplementation on weight (74). An analysis of five clinical trials involving 780 women reported a beneficial effect of calcium supplementation on body weight (80). Similarly, Shahar and cols. (32) reported a body weight reduction in a group with greater dietary calcium intake. Three systematic reviews have assessed the role of calcium supplementation on body weight. Onakpoya and cols. (81) found small but significant reductions in weight among overweight and obese individuals; however, the other two studies failed to confirm any correlation of dietary or calcium supplements with body weight (82,83).

#### Calcium and lipids

Reid and cols. (84) randomized 223 postmenopausal women to receive 1,000 mg/day of calcium citrate or placebo. These authors reported a 7% increase in high-density lipoprotein (HDL) levels at 12 months after the initiation of supplementation. Another study conducted by Reid reported no significant changes in lipids in men who received placebo or elemental calcium supplementation (600 to 1,200 mg/day) for 2 years (75). A decrease in triglyceride level following calcium supplementation has also been reported (76). In contrast, no significant change was observed in the serum lipids of 193 patients who were subjected to calcium supplementation (85).

## Dietary calcium supplementation *versus* medicinal calcium supplementation

As previously described, the studies that have assessed the effects of supplementary calcium on cardiovascular mortality have produced contradictory results (6-24). The results related to the intake of calcium-rich foods (*e.g*., milk, cheese, and yogurt) have demonstrated reduced mortality due to CVD (6,7,24) and reduced risks of MI (15,24) and stroke (21,23,24). These studies have also demonstrated neutral or beneficial profiles for factors such as atherosclerosis (26-28), blood pressure (29), plasma glucose (30,31) and body weight (32).

Additionally, the risk of nephrolithiasis, which is known to be elevated in patients taking calcium supplements (57), is minimized in patients who ingest calcium through dietary sources (33) because dietary calcium binds to oxalate inside the digestive tract to form a poorly absorbed complex, which reduces hyperoxaluria (which is responsible for the formation of most kidney stones). This effect does not seem to occur with the use of calcium supplements (86). Low-calcium food intake also increases the risk of nephrolithiasis because it facilitates the intestinal absorption of oxalate and consequently increases its renal clearance (86). Negative calcium balance leads to PTH secretion, which increases urinary calcium by stimulating bone resorption and may thus be an additional factor that triggers nephrolithiasis in these patients (87,88).

The effectiveness of supplementation still depends on the compliance of patients, which has been described as low among users of calcium supplements. Additionally, adverse gastrointestinal effects may limit adherence and are less common with dietary calcium nutrients (18,47-50).

Another benefit of dietary rather than medicinal supplementation is the minimization of the transient hypercalcemia that has been described in patients taking calcium supplements (63) and has been suggested to be the hypothetical cause of increased the cardiovascular risk in these patients (3,9).

In conclusion, several studies have demonstrated the efficacies of calcium supplementation alone or in combination with vitamin D supplementation in the development and maintenance of bone mass, osteoporosis treatment and the reduction of the risk of fractures. The intake of nutrients rich in calcium is a priority because such intake has the same benefits as medicinal supplementation in terms of bone health and provides other additional important nutrients, such as proteins, while minimizing the risk of side effects (e.g., nephrolithiasis and gastrointestinal intolerance) and low adherence to drug treatment. Thus, supplementation should be restricted to individuals who are unable to achieve the recommended daily nutritional intake.

Some authors have evaluated the risk-benefit ratio of calcium supplementation and suggested a possible increase in cardiovascular risk, which contrasts with other studies that have described neutral or even protective effects regarding cardiovascular complications. These hypotheses have not been conclusively proven or disproven, and the different study designs and primary outcomes prevent us from reaching conclusions. Calcium supplementation has been demonstrated to be safe with in terms of atherosclerosis, blood pressure, diabetes, body weight and dyslipidemia, and these factors are known to be correlated with the risk of cardiovascular complications. However, studies that have assessed the cardiovascular risk associated with the nutritional intake of calcium-rich foods have proven the safety of this approach; therefore, nutritional calcium intake should be prioritized.

Prescribing calcium and vitamin D supplementation only to those individuals who will benefit from such treatment, promoting the intake of calcium-rich foods, restricting supplementation to people who do not achieve the recommended daily requirements, and avoiding excessive supplementation by following the suggested recommendations (i.e., a maximum of 1,500 mg/day of calcium) are all useful practices for osteoporosis prevention and treatment and do not seem to increase the risk of cardiovascular complications.

Further studies aimed at assessing the risk-benefit ratios of calcium supplementation in different populations are needed to define calcium’s true relationship with cardiovascular outcomes.
